# Predictors of decline in self-reported health: addressing non-ignorable dropout in longitudinal studies of aging

**DOI:** 10.1007/s10433-017-0448-x

**Published:** 2017-12-02

**Authors:** Minna Genbäck, Nawi Ng, Elena Stanghellini, Xavier de Luna

**Affiliations:** 1 Department of Statistics, USBE, Umeå University, SE - 901 87, Umeå, Sweden; 2 Centre for Demographic and Ageing Research, Umeå University, Umeå, Sweden; 3 Unit of Epidemiology and Global Health, Department of Public Health and Clinical Medicine, Umeå University, Umeå, Sweden; 4 Department of Economics, University of Perugia, Perugia, Italy

**Keywords:** Longitudinal studies, Dropout, Sensitivity analysis, Chronic disease, Body mass index, SHARE

## Abstract

Predictors of decline in health in older populations have been investigated in multiple studies before. Most longitudinal studies of aging, however, assume that dropout at follow-up is ignorable (missing at random) given a set of observed characteristics at baseline. The objective of this study was to address non-ignorable dropout in investigating predictors of declining self-reported health (SRH) in older populations (50 years or older) in Sweden, the Netherlands, and Italy. We used the SHARE panel survey, and since only 2895 out of the original 5657 participants in the survey 2004 were followed up in 2013, we studied whether the results were sensitive to the expectation that those dropping out have a higher proportion of decliners in SRH. We found that older age and a greater number of chronic diseases were positively associated with a decline in self-reported health in the three countries studies here. Maximum grip strength was associated with decline in self-reported health in Sweden and Italy, and self-reported limitations in normal activities due to health problems were associated with decline in self-reported health in Sweden. These results were not sensitive to non-ignorable dropout. On the other hand, although obesity was associated with decline in a complete case analysis, this result was not confirmed when performing a sensitivity analysis to non-ignorable dropout. The findings, thereby, contribute to the literature in understanding the robustness of longitudinal study results to non-ignorable dropout while considering three different population samples in Europe.

**Electronic supplementary material:**

The online version of this article (10.1007/s10433-017-0448-x) contains supplementary material, which is available to authorized users.

## Introduction

As a subjective measure of overall health, self-reported health (SRH) provides an unspecific, yet comprehensive, measure of population health. Although researchers have no control over which aspects of health the individual emphasizes in their assessments of SRH (Jylhä 2009), the question itself can capture dimensions of health that more detailed health questions often miss. SRH has consistently been shown to be an important independent predictor of mortality, even after the analyses are adjusted with other more objective health indicators such as biological markers (Idler and Benyamini 1997; Kuhn et al. 2006).

Because SRH is subjective, several studies have suggested that there are differences in how different groups perceive their health, where for example women put more emphasis on disability than mortality compared to men (Deeg and Kriegsman 2003). There might also be differences between countries because cultural and linguistic factors might affect how the respondents answer the SRH question (Jylhä 2009; Jylhä et al. 1998). These differences in reporting SRH between population groups might lead to difficulties in comparing different studies.

A large number of cross-sectional studies have identified predictors of SRH. These predictors include socioeconomic variables such as income (Simons et al. 2013), education (Mirowsky and Ross 2008), occupation (Gueorguieva et al. 2009), social capital (Eriksson and Ng 2015; Giordano et al. 2012), and transition into divorce or widowhood (Liu 2012); lifestyle variables such as smoking, alcohol use, and physical inactivity (Hämmig et al. 2014; Rosenkranz et al. 2013); and other health-related variables such as body mass index (Wang and Arah 2015). In summary, these earlier results indicate that better socioeconomic status and healthier lifestyle are positively associated with SRH, and either very low or very high body mass index is negatively associated with SRH.

Evidence for predictors of change in SRH over time has also been provided in longitudinal studies (Cullati et al. 2014; Svedberg et al. 2005). These predictors include age; sex (Rohlfsen and Kronenfeld 2014); socioeconomic level, including income (Giordano and Lindstrom 2010), education level (Lee et al. 2012), and labor market participation (Gueorguieva et al. 2009); health, including both physical and psychological health (Ayyagari et al. 2012; Verropoulou 2012); cognitive status; social capital (Giordano and Lindstrom 2010); and lifestyle such as alcohol consumption and physical activity (Sargent-Cox et al. 2014).

Dropout occurs in most follow-up studies, especially in studies of aging. Most missing data methods assume an ignorable dropout mechanism, i.e., that dropout is unrelated to the outcome given some measured covariates. It is well known that if this ignorability assumption is not fulfilled, the results can be biased (Little and Rubin 2002). Moreover, an ignorable dropout mechanism is unrealistic in follow-up studies where the outcome of interest is related to health, since then one expect dropout to be directly dependent on deteriorating health; see, e.g., Josefsson et al. (2016) for a practical example in a study of cognitive decline. Methods taking into account non-ignorable dropout are either based on strong identifying assumptions and/or using extra information like instrumental variables (Molenberghs et al. 2015), or, as we propose herein, estimating bounds under milder assumptions (Genbäck et al. 2015; Vansteelandt et al. 2006).

Even though there is a vast literature covering transitions in SRH, there are few covering more than one country at a time and, importantly, even fewer consider possible biases due to non-ignorable dropout. The main objectives of this study were to identify predictors of decline in SRH in older populations (50 years or older) in three European countries, while showing that evidence obtained from the data may depend on whether we ignore dropout or whether we account for the probable situation that SRH decliners are overrepresented among those dropping out at follow-up. We focused on three countries from different parts of Europe, the north (Sweden), west (Netherlands), and south (Italy), in order to corroborate results across different contexts and sampling schemes.

## Methods

### Design, study sample, and data collection

We analyzed data from the Survey of Health, Ageing and Retirement in Europe (SHARE). SHARE is a panel survey conducted on a random sample from the population aged 50 or older in European countries. The questionnaires were administered through face-to-face computer-assisted interviews. The first wave (baseline) started in 2004, and the last follow-up, wave 5, was conducted in 2013. The target population in each country was all residents born in 1954 or earlier, speaking the native language, and not living in prisons and similar institutions; as well as their spouses, irrespective of their age. Since the sampling process in SHARE was on a household level, all individuals in a household (almost exclusively one person or a man and a woman) were included in the sample. If both women and men were analyzed together, the samples would not have independent observations; therefore, we stratified on sex. We did not include the in-between waves since we did not want to make stronger assumptions than we currently do. Using in-between waves would imply the use of further parametric assumptions, and the sensitivity analysis would thereby become more complex. We took the two furthest observation time points to allow for larger declines. In this study, we focused on the SHARE sample in Sweden, the Netherlands, and Italy. The sampling procedure differed between countries, and thus the countries were analyzed separately. In Sweden, stratified simple random sampling from the population register was used, and in the Netherlands and Italy multi-stage sampling from regional registers (where some regions chose not to participate) was used. More details about the sampling process and target population have been published elsewhere (Börsch-Supan and Jürges 2005).

The response rate at baseline was 41% in Sweden, 54% in the Netherlands, and 44% in Italy (Börsch-Supan and Jürges 2005). Of the study sample that participated at baseline, the 9-year follow-up response rate in 2013 was 51, 48, and 57%, respectively. We were not able to separate death from non-response at follow-up, since information about who was alive or dead at follow-up was only available for 16% of the individuals who dropped out.

**Fig. 1:**
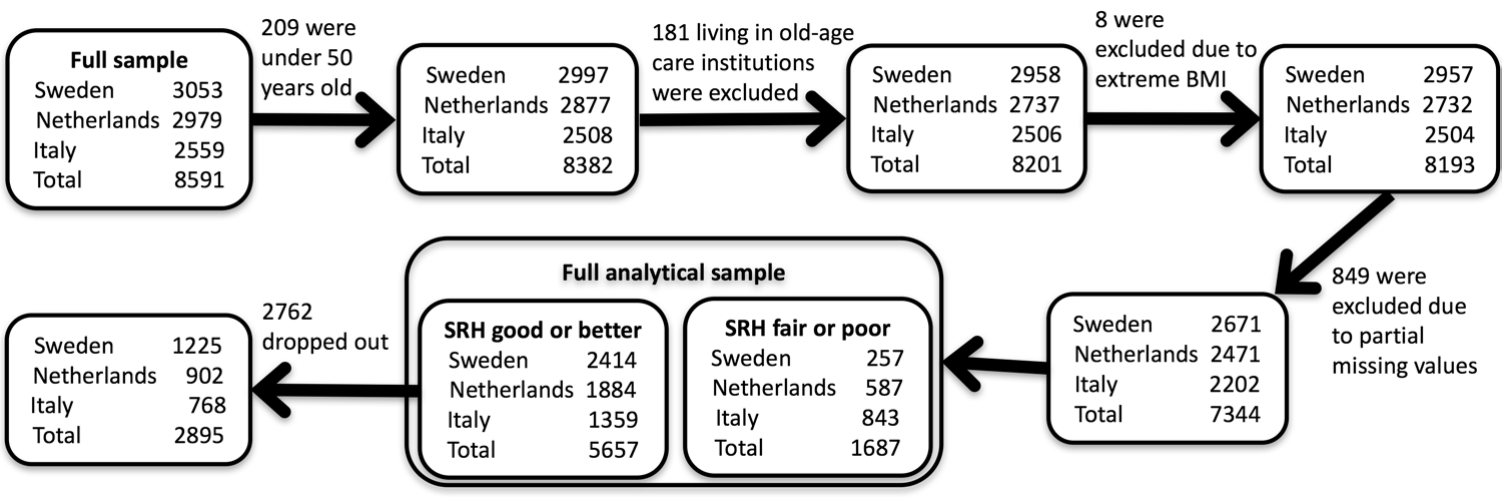
Flowchart of stages of inclusion in the study: the size of the study sample at baseline (box labeled “SRH good or better”) and at follow-up

The study sample was further limited to individuals born 1954 or earlier. As individuals living in old-age care institutions in Italy were not sampled, we excluded the individuals living in old-age care institutions in the other two countries to make the samples comparable. Individuals with partial missing values, or outlier values on body mass index (lower than 15 or larger than 50) were removed from the sample. Since individuals in poor health cannot decline according to our definition (see next section), we limit our study to individuals who reported “Good health” at baseline (year 2004). Figure 1 shows the exact numbers of individuals included at baseline and the numbers remaining after dropout at follow-up.

### Variables

#### Outcome variable: decline in self-reported health (SRH)

The survey asked the respondents about their SRH using the question “Would you say your health is: excellent, very good, good, fair or poor?” We dichotomized these answers into the two categories of “Good health” (including the responses “Excellent,” “Very good,” or “Good”) and “Poor health” (“Fair” or “Poor”). We wanted to investigate predictors of decline in SRH to the potentially harmful “Poor health” category; since only few answered poor at follow-up, we decided to include fair into the “Poor health” category. We defined the outcome to reflect the transition of SRH from baseline to the last follow-up as a binary variable taking the value of 1 if the respondents reported “Good health” at baseline and “Poor health” at the follow-up, and 0 otherwise.

#### Covariates

We included measures of cognitive function, health, lifestyle, and socioeconomic status at baseline in 2004 as potential predictors (SHARE Release Guide 2.6.0, Wave 1 and Wave 2.).

The measures of cognitive function consisted of mathematical performance based on a numerical test with possible scores of 1 to 5 that were dichotomized into two levels, good (scores of 4 or 5) and bad (scores of 3 or less), and the ability to state the correct date, month, year, and day of the week dichotomized into 1 if all answers were correct and 0 otherwise.

The health-related variables included the number of self-reported chronic diseases (heart attack or other heart problems; high blood pressure; high blood cholesterol; stroke; diabetes; chronic lung disease; asthma; arthritis, osteoporosis; cancer; peptic ulcer; Parkinson disease; cataracts; hip fracture; or other conditions); the number of self-reported problems with mobility (walking 100 meters; sitting for about two hours; getting up from a chair after sitting for long periods; climbing several flights of stairs without resting; climbing one flight of stairs without resting; stooping, kneeling, or crouching; reaching or extending your arms above shoulder level; pulling or pushing large objects like a living room chair; lifting or carrying weights over 5 kilos; or picking up a small coin from a table); self-reported symptoms of depression (measured using twelve questions based on the Euro-D scale developed by Prince et al. (1999)—the respondents were considered depressed if they answered positively for three or more symptoms); maximum grip strength (in kg, maximum of 4 measures using a dynamometer); self-reported limitation due to health problems in normal activities (using the global activity limitation indicator); and body mass index (kg/m^2^, derived from self-reported height and weight), categorized into normal weight (< 25, reference level), overweight (25–29.9) and obesity (≥ 30).

The lifestyle variables included smoking (categorized as current smoker, never smoked daily for at least 1 year, stopped smoking), high alcohol use (whether respondents drank more than two glasses of alcohol at least 5–6 days a week), and being inactive (if respondents answered at most 1–3 times a month on both of the questions “How often do you engage in vigorous physical activity, such as sports, heavy housework, or a job that involves physical labor?”or “How often do you engage in activities that require a low or moderate level of energy such as gardening, cleaning the car, or doing a walk?”).

The socioeconomic variables consisted of education level (based on ISCED-97 code and dichotomized into “at most lower secondary education”—compulsory school—or “upper secondary education or higher”) and if the household was able to make ends meet (dichotomized into “with great or some difficulty” or “fairly easily or easily”).

We also controlled for the respondent’s age and a variable that indicated whether the respondent was randomized to answer the self-perceived health question at the beginning or at the end of the questionnaire during the baseline interview.

### Statistical analysis

For each country, men, and women separately, the outcome was modeled using logistic regression assuming that the dropout mechanism (indicator variable taking the value 1 if an individual participates at the follow-up and 0 if they drop out) and outcome were independent when conditioning on the observed covariates (missing at random/ignorable dropout).

We carried out a sensitivity analysis to investigate how sensitive our results were to the assumption of ignorable dropout. The sensitivity analysis that we used was adapted from the method proposed in Genbäck et al. (2015). It is described in detail together with a simplified example illustrating the ideas and concept behind such an analysis in the supplemental material. Briefly, the sensitivity analysis is based on explaining both dropout and the outcome with two regression models with the same covariates, whose two error terms are allowed to be correlated (with correlation ρ). If dropout is ignorable, then the dropout and outcome dependence is due only to the covariates included in the regression models, i.e., the error terms in the two regression models are uncorrelated. If the outcome is still associated with dropout after controlling for the covariates (i.e., ρ is different from zero), dropout is called non-ignorable. This correlation (ρ) is used as a sensitivity parameter, to investigate the consequence of departure from ignorable dropout. Indeed, if ρ = 0, then we have ignorable dropout, while if ρ = − 1, then all individuals dropping out have declined to poor SRH, and if ρ = 1, all individuals dropping out have remained in good SRH. In this paper, only negative correlations were considered, ρ∈[− 0.8, 0], in accordance with the realistic assumption that dropping out is in part related to poor health. We derived uncertainty intervals (the analog of confidence intervals but assuming that ρ∈[− 0.8, 0] instead of ρ = 0) that take into account uncertainty in ρ, and report them together with 95% confidence intervals (assuming ρ = 0) in the result tables. All the analyses were performed in R-statistical software (R Core Team 2015), and the code is freely available from the authors upon request.

## Results

### Description of the study subjects

The baseline characteristics of female and male respondents are summarized in Tables 1 and 2, respectively. There were slightly more female than male respondents in all countries studied.

**Table 1:** Descriptive statistics for the female respondents with good or better self-rated health at baseline

Variables	Sweden (*N* = 1235)		Netherlands (*N* = 985)		Italy (*N* = 682)	
	Value	(95% CI)	Value	(95% CI)	Value	(95% CI)
Baseline variables						
Mean age in years	63.8	(63.3, 64.4)	61.8	(61.2, 62.3)	61.9	(61.3, 62.6)
% who responded to the SRH question at the beginning of the interview	47.0	(44.2, 49.9)	51.8	(48.6, 55.0)	47.7	(43.8, 51.5)
Socioeconomic variables						
% with high education level	51.8	(49.0, 54.7)	39.6	(36.5, 42.7)	28.0	(24.6, 31.4)
% make ends meet fairly easily or easily	80.2	(78.0, 82.5)	83.2	(80.9, 85.6)	40.5	(36.7, 44.2)
Cognitive function variables						
% with good numeracy test	50.8	(47.9, 53.6)	50.3	(47.1, 53.4)	25.1	(21.8, 28.4)
% with good date orientation	93.1	(91.7, 94.6)	87.7	(85.6, 89.8)	89.6	(87.3, 91.9)
Health-related variables						
Overweight, 25 ≤ body mass index < 30	35.8	(33.1, 38.5)	36.8	(33.7, 39.8)	37.0	(33.3, 40.6)
Obesity, body mass index ≥ 30	11.8	(10.0, 13.7)	13.2	(11.0, 15.4)	14.2	(11.5, 16.9)
Mean number of chronic diseases	1.33	(1.26, 1.40)	0.97	(0.90, 1.05)	1.15	(1.06, 1.24)
Mean number of mobility problems	0.97	(0.88, 1.05)	0.68	(0.60, 0.76)	0.92	(0.81, 1.04)
% with depression	35.3	(32.6, 38.0)	29.1	(26.2, 32.0)	41.6	(37.9, 45.4)
Mean of maximum grip strength in kg	27.6	(27.3, 28.0)	29.8	(29.4, 30.2)	26.0	(25.6, 26.5)
% with limitation in normal activities	37.9	(35.1, 40.7)	35.4	(32.4, 38.5)	21.4	(18.3, 24.5)
Lifestyle variables						
% who stopped smoking	31.7	(29.1, 34.4)	29.8	(26.9, 32.8)	16.1	(13.3, 18.9)
% who were current smokers	18.5	(16.3, 20.8)	21.2	(18.6, 23.8)	16.4	(13.6, 19.3)
% with high alcohol usage	1.05	(0.47, 1.63)	13.7	(11.5, 15.9)	12.9	(10.3, 15.5)
% with physical inactivity	3.32	(2.30, 4.34)	4.26	(2.98, 5.55)	17.0	(14.1, 19.9)
Follow-up variables						
% who were lost-to follow-up	47.7		49.6		43.1	
% with decline in SRH among those who were followed up	24.0		23.0		41.8

**Table 2:** Descriptive statistics for the male respondents with good or better self-rated health at baseline

Variables	Sweden (*N* = 1179)		Netherlands (*N* = 899)		Italy (*N* = 677)	
	Value	(95% CI)	Value	(95% CI)	Value	(95% CI)
Baseline variables						
Mean age in years	64.7	(64.1, 65.3)	62.6	(62.0, 63.2)	63.4	(62.7, 64.0)
% who responded to the SRH question at the beginning of the interview	49.0	(46.1, 51.9)	48.6	(45.3, 51.9)	45.6	(41.8, 49.5)
Socioeconomic variables						
% with high education level	47.8	(44.8, 50.7)	57.8	(54.5, 61.1)	29.5	(26.0, 33.0)
% make ends meet fairly easily or easily	84.1	(81.9, 86.2)	84.3	(81.9, 86.7)	39.4	(35.7, 43.2)
Cognitive function variables						
% with good numeracy test	63.1	(60.3, 65.9)	72.0	(69.0, 75.0)	36.0	(32.4, 39.7)
% with good date orientation	88.3	(86.4, 90.2)	85.5	(83.2, 87.9)	86.9	(84.3, 89.5)
Health-related variables						
Overweight, 25 ≤ body mass index < 30	48.5	(45.6, 51.4)	50.9	(47.6, 54.3)	53.9	(50.1, 57.7)
Obesity, body mass index ≥ 30	12.6	(10.7, 14.6)	10.6	(8.5, 12.6)	14.8	(12.0, 17.5)
Mean number of chronic diseases	1.32	(1.25, 1.39)	0.88	(0.81, 0.94)	1.07	(0.99, 1.16)
Mean number of mobility problems	0.54	(0.48, 0.61)	0.30	(0.25, 0.35)	0.45	(0.38, 0.53)
% with depression	17.0	(14.9, 19.2)	16.7	(14.2, 19.2)	25.3	(21.9, 28.6)
Mean of maximum grip strength in kg	45.6	(45.0, 46.1)	47.1	(46.5, 47.7)	42.0	(41.3, 42.8)
% with limitation in normal activities	33.6	(30.8, 36.3)	24.9	(22.0, 27.8)	14.6	(11.9, 17.3)
Lifestyle variables						
% who stopped smoking	45.6	(42.7, 48.5)	50.2	(46.8, 53.5)	38.0	(34.2, 41.7)
% who were current smokers	13.7	(11.7, 15.7)	26.3	(23.3, 29.2)	23.9	(20.6, 27.2)
% with high alcohol usage	3.05	(2.05, 4.06)	24.9	(22.0, 27.8)	43.9	(40.1, 47.7)
% with physical inactivity	3.31	(2.27, 4.35)	4.56	(3.17, 5.95)	13.7	(11.1, 16.4)
Follow-up variables						
% who were lost-to follow-up	50.9		54.8		43.9	
% with decline in SRH among those who were followed up	21.9		24.4		35.8	

The Swedes were slightly older (confidence intervals in Tables 1 and 2 for Swedish respondents did not overlap with those of Dutch and Italian respondents) and reported more chronic diseases, and the men reported more limitations in normal daily activities due to health problems than their counterparts in the Netherlands and Italy. A high proportion of the Swedish women had high education level and performed well in the cognitive tests. The Swedes also had a lower proportion of individuals with high alcohol use, and the Swedish men had a lower proportion of current smokers than the other countries.

The Dutch, on the other hand, reported fewer chronic diseases and had a higher grip strength compared to their counterparts in Sweden and Italy. Also, a lower proportion of the Dutch women had depressive symptoms. The Dutch men had higher education level and a higher proportion with a good score in the numerical test compared to the men in Sweden and Italy.

The Italians had the lowest proportion of respondents with high education level and good score on the numerical test, and a higher proportion of individuals with difficulty to make ends meet compared to Sweden and the Netherlands. Even though the Italians had a higher proportion of physically inactive individuals (17 and 14% among women and men, respectively, versus less than 5% in the other two countries), they had a lower proportion of individuals who felt limited in their daily activities due to health problems. The Italians also reported more depressive symptoms and had the lowest grip strength. In the longitudinal analysis, we observed a higher proportion of Italians reporting a decline in SRH (42% among women and 36% among men) compared to at most 24% in the other two countries.

### Predictors of decline in SRH

This study highlights age and number of chronic diseases as two predictors of decline in SRH across countries (Tables 3 and 4). Female respondents in all three countries had a higher risk of decline in SRH with increasing age.

**Table 3:**
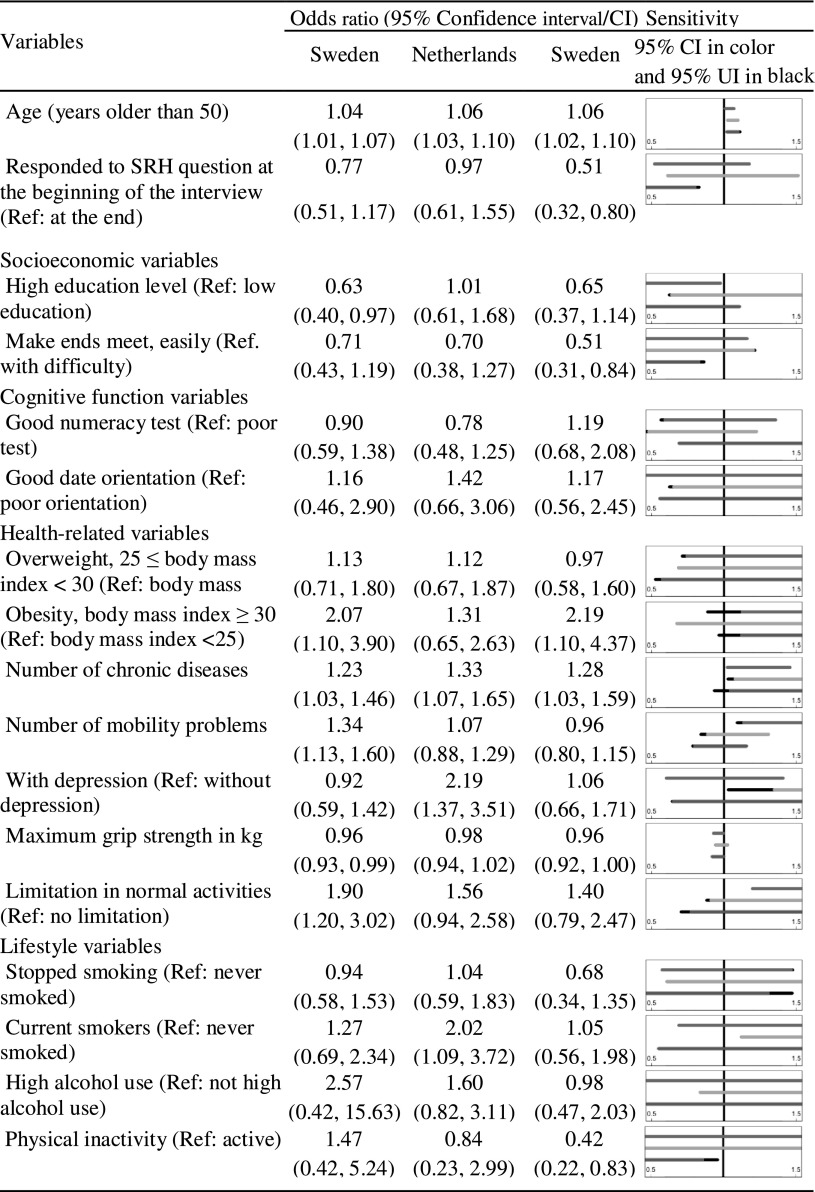
Odds ratios and 95% confidence intervals from the multiple logistic regressions modeling decline in SRH against all covariates for females

Figure panels show the 95% confidence interval of OR (in blue, orange, and green for Sweden, the Netherlands, and Italy, respectively) and uncertainty intervals (UI, in black). The uncertainty intervals are derived assuming that the dropout mechanism is positively correlated with decline in SRH (correlation between − 0.8 and 0). (Color table online)

**Table 4:**
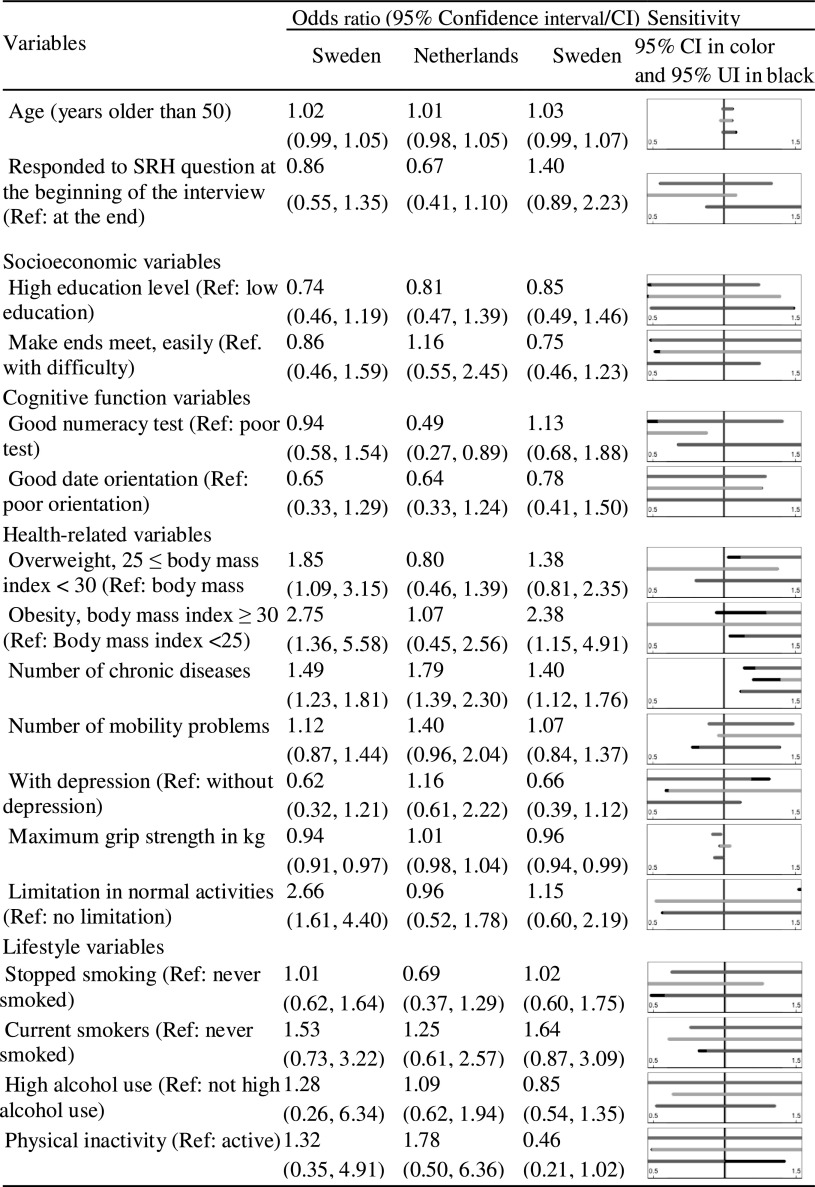
Odds ratios and 95% confidence intervals from the multiple logistic regressions modeling decline in SRH against all covariates for males

Figure panels show uncertainty intervals (in black) and a confidence interval (in blue, orange, and green for Sweden, the Netherlands, and Italy, respectively). The uncertainty intervals are derived assuming that the dropout mechanism is positively correlated with decline in SRH (correlation between − 0.8 and 0). (Color table online)

For each additional self-reported chronic condition at baseline, the odds of decline in SRH increased, ranging from 23% (odds ratio (OR) 1.23 95% confidence interval (CI) [1.03–1.46]) among the Swedish women to 79% (1.79 [1.39–2.30]) among the Dutch men. For the Italian women, the association between number of chronic diseases and decline in SRH was sensitive to non-ignorable dropout because the 95% CI assuming ignorable dropout ([1.03–1.59]) did not contain 1, but the corresponding uncertainty interval ([0.93–1.59], shown in black in Table 3), allowing for non-ignorable dropout did.

The complete case analysis indicates that being obese is positively associated with decline in SRH in Sweden (2.07 [1.10–3.90] among women and 2.75 [1.36–5.58] among men) and Italy (2.19 [1.10–4.37] among women and 2.38 [1.15–4.91] among men). The corresponding uncertainty intervals are [0.89–3.90], [0.95–5.58], [0.97–4.37] and [1.04–4.91]. In fact, all uncertainty intervals except for the interval for Italian males include 1. Therefore, an association between obesity and decline in SRH is not supported by the sensitivity analysis, and we conclude that this result is sensitive to non-ignorable dropout. No significant association between being obese and decline in SRH was found for men and women in the Netherlands.

Self-reported limitations in normal activities due to health problems were associated with decline in SRH among the Swedish women (1.90 [1.20–3.02]) and men (2.66 [1.61–4.40]). High maximum grip strength protected against decline in SRH in Sweden (0.96 [0.93–0.99] among women and 0.94 [0.91–0.97] among men) and Italy (0.96 [0.92–1.00 among women and 0.96 [0.94–0.99] among men). These results were not sensitive to non-ignorable dropout.

Somewhat surprisingly, we found that being inactive seemed to be protective against decline in SRH among the Italian women (0.42 [0.22–0.83]). However, this effect was not apparent in the other country and sex combinations.

## Discussion

The objective of this study was to find predictors of declining self-reported health in older populations (50 years or older) in Sweden, the Netherlands, and Italy. Since we suspected that dropout may be positively related to decline in self-reported health, we also aimed at performing a sensitivity analysis of the assumption of ignorable dropout. We found that, after taking non-ignorable dropout into account, the number of chronic diseases was a predictor in all countries (except for Italian women) and maximum grip strength was a predictor associated with decline in SRH in Sweden and Italy. The complete case analysis, i.e., ignoring dropout, indicates that obesity is a predictor of decline in SRH among Swedish and Italian men and women. However, our sensitivity analysis showed that three out of four associations that were significant in the complete case analysis were sensitive to non-ignorable dropout. This is in part due to there being a higher proportion of obese individuals among those that drop out than those that participate at follow-up. We concluded that there was not enough evidence in the data to consider obesity a predictor of SRH decline. This demonstrates that taking into account non-ignorable dropout, in our case implying that those with poor health may have a larger propensity to dropout, impacts the results of our longitudinal study. These results may be contrasted with Rohlfsen and Kronenfeld (2014) who found that being overweight was associated with decline in SRH for men in the USA. However, other studies did not confirm high body mass index to be a predictor of declining health (Ayyagari et al. 2012; Lee et al. 2012). The results obtained for women and men are qualitatively similar.

Our study showed that age is a predictor of decline in SRH, even when accounting for non-ignorable dropout, which is in line with previous research (Ayyagari et al. 2012; Cullati et al. 2014; Lee et al. 2012). Multimorbidity with chronic diseases has been shown to be a predictor of decline in SRH (Hsu 2015; Lee et al. 2012), and our results confirm these findings. This is also in line with Ayyagari et al. (2012) who have identified diabetes, congestive heart failure, and angina as predictors for decline in SRH. In contrast, Rohlfsen and Kronenfeld (2014) investigated the association between several chronic diseases and change in SRH, and identified only arthritis as a predictor for health decline among women.

Some predictors were found to be significant only in some of the country/sex combinations. For instance, high maximum grip strength was found to be a predictor associated with non-decline in SRH for both men and women in Sweden and Italy, and self-reported limitations due to health problems in normal activities were associated with decline in SRH for both men and women in Sweden. The variations between countries might be due to a lack of statistical power, to the different sampling schemes, or to cultural differences in the answers provided to the outcome question “How is your health?” For instance, individuals in different countries might put emphasis on different aspects of health in their self-assessment, or there might be linguistic/translation issues and the SRH question and answer alternatives might not be interpreted in comparable ways (Jylhä 2009; Jürges 2007).

We had fairly low response rates at baseline (41% in Sweden, 54% in the Netherlands, and 44% in Italy). Thus, the results obtained in this study based on the respondents at baseline might not be generalizable to those that did not respond. If the two groups differ, we have no means to know how this would change the results. For 84% of the dropout individuals, we do not have information on whether the individuals were alive or not at follow-up. This information could have been used to make a distinction between dropout due to death or other reasons (Josefsson et al. 2016). However, we do not expect that a sensitivity analysis where death and other dropout causes are distinguished would yield different conclusions. For instance, a sensitivity analysis removing the 439 individuals known to be deceased yielded similar results (results not shown).

Even though there is a vast literature covering transitions in SRH, there are only few covering more than one country at a time, allowing to corroborate results in different population and sampling schemes. Moreover, most published longitudinal studies of aging assume that dropout at follow-up is ignorable (independent of the outcome) given a set of observed characteristics at baseline. However, this assumption is not realistic when a health outcome is of interest and dropout is expected to be related to health conditions. Our study is, up to our knowledge, the first one on predictors of changes in SRH that considers non-ignorable dropout. The study demonstrates that conclusions can indeed change when dropout mechanisms and SRH decline are allowed to be dependent on each other. The methodology presented to take into account non-ignorable dropout can be applied to any study with binary outcome observed at two time points and where non-ignorable dropout is expected.

## Electronic supplementary material

Below is the link to the electronic supplementary material.

Supplementary material 1 (PDF 192 kb)
