# Supplementary material for: Predictors of decline in self-reported health: addressing non-ignorable dropout in longitudinal studies of aging
Source: Eur J Ageing. 2017 Dec 2;15(2):211–20. doi: 10.1007/s10433-017-0448-x (PMC5971030; doi:10.1007/s10433-017-0448-x)
Supplement: Supplementary file 1 — Supplementary material 1 (PDF 192 kb) [file 10433_2017_448_MOESM1_ESM.pdf]

Supplemental material: Predictors of decline in self-reported health: addressing non-ignorable dropout in longitudinal studies of ageing

Sensitivity analysis

We propose a sensitivity analysis to the assumption of ignorable dropout (which is made when doing complete case analyses). We start with a simple example to introduce the concept of sensitivity analysis, before we present formally the method proposed.

Example 1: Estimating the proportion of decliners

Assume we are interested in estimating the proportion of individuals declining from good health to poor health ($\mathbf{p}$) from baseline to follow-up among the Swedish females. Out of the 1235 individuals responding at baseline only 646 participate at follow up (and 589 drop out). Out of these 646, 155 are decliners and 491 are not decliners. If we point estimate the proportion of decliners by using only the complete cases (i.e. the 646 that participate at follow-up), we get $\hat{\mathbf{p}} = \frac{155}{646} = 0.24$. This estimate is only unbiased if we assume that the dropout mechanism is ignorable.

The information we have from the data, however, is that between 155 and $(155 + 589) = 744$ out of the 1235 are decliners, since we do not know how many of the 589 non-respondents have declining SRH. That is, without further assumptions on the missing data, we can derive bounds for $\hat{\mathbf{p}}$, $\frac{155}{1235} = 0.13 \leq \hat{\mathbf{p}} \leq 0.60 = \frac{744}{1235}$, we call the interval $[0.13, 0.60]$ a worst case scenario bound of $\hat{\mathbf{p}}$.

Since we only observe a sample and not the entire underlying population, we want to derive a 95% confidence interval for $\mathbf{p}$. Assuming ignorable dropout the confidence interval centered around $\hat{\mathbf{p}} = \frac{155}{646} = 0.24$ is $[0.21, 0.27]$. The analog of a 95% confidence interval but centered around the interval $[0.13, 0.60]$ is called herein a 95% uncertainty interval. We

derive the 95% uncertainty interval, [0.11, 0.63], from the worst case senario bound above by adding confidence bounds to the lower and upper end of the worst case scenario bound. Note that the uncertainty interval contain the confidence interval. This is natural since this uncertainty interval is derived without assumptions on the missing data. Hence the uncertainty due to missing data is incorporated into a wider interval.

Let us now introduce as a sensitivity parameter the proportion of decliners among the non-respondents, $\mathbf{p}_{Z=0}$. Ignorable dropout can now be formulated as $\mathbf{p}_{Z=0} = \mathbf{p}_{Z=1}$, i.e. the proportion of decliners is the same among the non-respondents and respondents. Alternatively, the no assumption interval estimate from above corresponds to letting the probability vary freely between 0 and 1, i.e. $\mathbf{p}_{Z=0} \in [0, 1]$. Hence, we retrieve our earlier results: $\hat{\mathbf{p}} = 0.24$ if $\mathbf{p}_{Z=0} = \mathbf{p}_{Z=1}$ and $\hat{\mathbf{p}} \in [0.13, 0.60]$ if $\mathbf{p}_{Z=0} \in [0, 1]$. Thus, knowledge (or lack thereof) on the sensitivity parameter leads to different inference for $\mathbf{p}$. Suppose that we have subject matter knowledge that dropout is related to poor health. In such a case, we can assume that $\mathbf{p}_{Z=0} \geq \mathbf{p}_{Z=1}$ and we can estimate bounds for $\hat{\mathbf{p}}$ as $[0.24, 0.60]$. Note that the lower bound is $\hat{\mathbf{p}}_{Z=1} = 0.24$ and the upper bound the same as in the worst case scenario bound given above. If we derive the corresponding 95% uncertainty interval we get [0.21, 0.63].

Model and method

In Example 1 the interest was in estimating the proportion of decliners from good SRH to poor SRH. We now generalize the sensitivity analyses presented above to the estimation of predictors coefficients in a regression for binary outcome (declining/not declining), where the latter outcome is missing for those dropping out. If the dropout mechanism is ignorable, a well specified logistic or probit regression analysis of the outcome against the covariates using only the complete cases (the ones participate at follow-up) would yield unbiased estimates of the regression coefficients. However, if the dropout mechanism is not ignorable the estimates from a complete case analysis are biased. In order to allow for the dropout mechanism to be non-ignorable, we model both the outcome and the dropout mechanism with probit regression models. This is formalised below.

Let Y be a binary outcome and Z be an indicator variable (the dropout mechanism) that take the value 1 if Y is observed and 0 otherwise. We model these variables by letting $Z = I(Z^* \geq 0)$ and $Y = I(Y^* \geq 0)$ where $I(v)$ is the indicator function taking value 1 if v

is true and 0 otherwise, and (Y^*, Z^*) are two latent random variables such that:

$$\begin{cases} Z^* &= \boldsymbol{\delta}^T \mathbf{x} + \varepsilon, \\ Y^* &= \boldsymbol{\beta}^T \mathbf{x} + u, \end{cases} \tag{1}$$

where ε and u are two jointly Gaussian random variables, with standardized marginals and correlation ρ, and $\mathbf{x}$ is a k-dimensional covariate vector with the first element fixed to one (intercept). With this model dropout is ignorable if $\rho = 0$ and non-ignorable if $\rho \neq 0$, i.e. ρ is here the sensitivity parameter. This model and the resulting sensitivity analysis is a generalization of the approach presented in Genbäck et al. (2015), where the observed outcome was continuous. A similar approach was also presented in Copas and Li (1997) and Stingo et al. (2011).

In order to estimate the regression parameters we use maximum likelihood for the model in (1), see details at the end of this document. Maximization with respect to all parameters without further assumptions is unstable, since the likelihood function is flat in the ρ dimension; there is no information in the data on ρ. Therefore, ρ is instead used as a sensitivity parameter, and varied in order to look at the consequence on $\hat{\beta}$, the estimator of the parameter of interest. We first assume ρ known and fixed and derive the estimates $\hat{\boldsymbol{\delta}}(\rho)$ and $\hat{\boldsymbol{\beta}}(\rho)$ by maximization of the joint log-likelihood (2), given ρ. Sampling variation is taken into account by computing standard errors (from the inverse of the Fisher information matrix) and then constructing confidence intervals for $\boldsymbol{\delta}$ and $\boldsymbol{\beta}$ given ρ.

The uncertainty interval (UI) for $\boldsymbol{\beta}$ is the union of all the confidence intervals for $\boldsymbol{\beta}$ obtained with ρ varying in a given interval $[a, b]$ (Vansteelandt et al. 2006). If we denote the lower and the upper bound of the confidence interval for β_j (element of $\boldsymbol{\beta}$) with $\mathrm{LCI}^j(\rho)$ and $\mathrm{UCI}^j(\rho)$ for a fixed ρ then:

$$UI = (min_{\rho\in[a,b]}\mathrm{LCI}^j(\rho)\,;\ max_{\rho\in[a,b]}\mathrm{UCI}^j(\rho)).$$

Not that in the sensitivity analysis the model used for the binary outcome is a probit. Another popular specification is based on the logit link. Probit and logit inference seldom disagree, and the probit coefficients could be used to approximate logit with very high precision (Demidenko 2004; pp. 334-337). Thus, we argue that even if logit models are fitted (as we do in the paper) to obtain odds ratios, the probit-based sensitivity analysis is still relevant.

Example 2: Obesity as predictor of decline

Let us look at the sensitivity analysis for the coefficient for body mass index ≥ 30 (Obesity) for both women and men in the three countries, (Figure 1 - 8). In the sensitivity analysis we assume a negative correlation between ε and u ($[a, b] = [-0.8, 0]$), which is in line with a belief that dropout is related to poor health. Figure 1 - 3 and 5 - 7 illustrates the maximum likelihood estimates for the element of $\boldsymbol{\beta}$ that corresponds to Obesity for the different values of ρ, $\hat{\boldsymbol{\beta}}_j(\rho)$, the corresponding confidence intervals (CI) and resulting uncertainty interval (UI). The colored interval is the confidence interval assuming ignorable dropout ($\rho = 0$), i.e. identical to a confidence interval from a complete case analysis. The black dashed intervals are the confidence intervals assuming $\rho = -0.1, -0.2, ..., -0.8$. The uncertainty interval is the black unbroken interval (union of the colored and all the dashed intervals).

In Figure 1 we can see that assuming ignorable dropout ($\rho = 0$, same results as a complete case analysis) Obesity is significant at the 5 % level for Swedish women (the blue confidence interval does not contain 0). The coefficient estimate decrease with negative ρ, and the confidence intervals assuming $\rho = -0.6$, -0.7 and -0.8 all include 0 (dashed lines). Consequently the uncertainty interval assuming that $\rho \in [-0.8, 0]$ includes 0 and we say that the conclusions from a complete case analysis is sensitive to non-ignorable dropout. Similar results are found for Italian women and Swedish men, see Figure 3 and 5. For Italian men the coefficient estimate also decreases when $\rho < 0$, but the uncertainty interval does not contain 0, which suggests that the association in the complete case analysis might be overestimated. In summary the sensitivity analysis suggests that the significance of Obesity as a predictor of decline in SRH cannot be trusted.

Figure 4 and 8 contain a summary of Figure 1 - 3 and 5 - 7 respectively. At the top of Figure 4 and 8 we see in color (blue Sweden, orange Netherlands, green Italy) confidence intervals (assuming ignorable dropout) for Obesity from the probit regression. The black continuations of the coloured intervals show the uncertainty intervals assuming $\rho \in [-0.8, 0]$. Below in Figure 4 and 8 we have translated the intervals from a probit scale into an approximated odds ratio scale in order to simplify comparison with the odds ratios in the manuscript, this is presented in the manuscript in Table 3 and 4.

Technical details

We now explain how to derive the log likelihood of the model (1). Note that, we can only observe Z and ZY from the data, and never Z^* or Y^*. More specifically, three types of

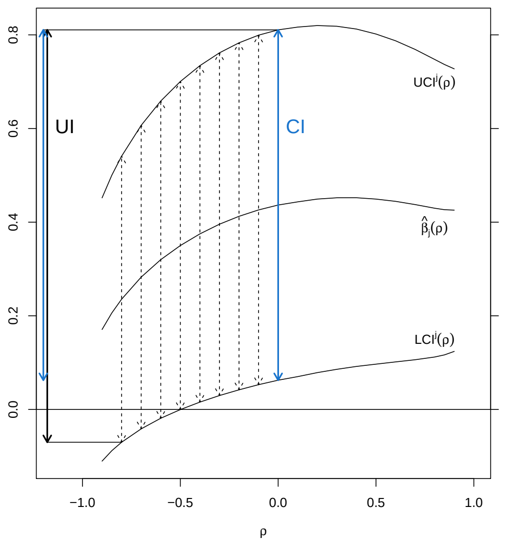

Figure 1: Sensitivity analysis ($\alpha = 5\%$, $\rho \in [-0.8, 0]$) for Obesity, Swedish women (see text).

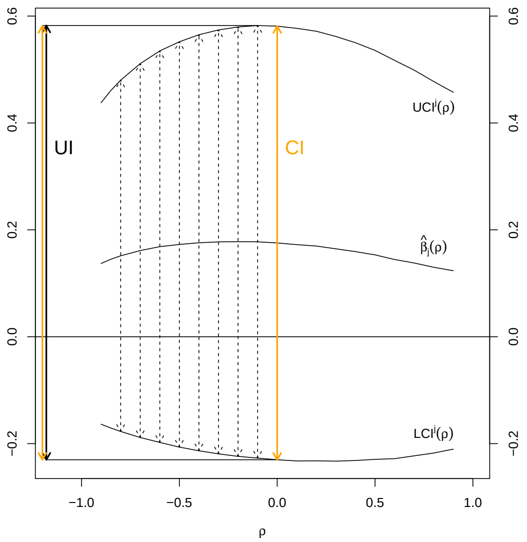

Figure 2: Sensitivity analysis ($\alpha = 5\%$, $\rho \in [-0.8, 0]$) for Obesity, Dutch women (see text).

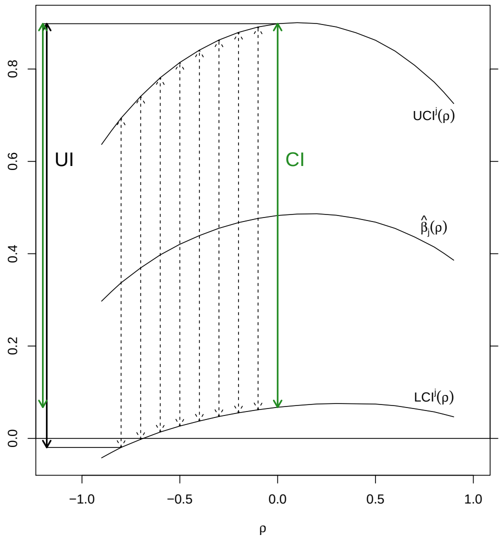

Figure 3: Sensitivity analysis ($\alpha = 5\%$, $\rho \in [-0.8, 0]$) for Obesity, Italian women (see text).

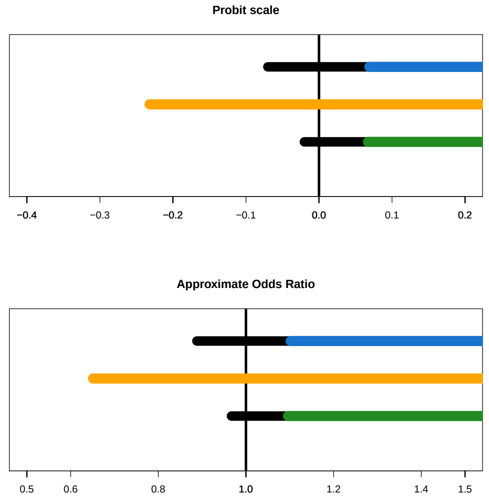

Figure 4: Confidence interval assuming ignorable dropout (blue Sweden, orange Netherlands and green Italy) drawn over the uncertainty interval assuming $\rho \in [-0.8, 0]$ in black.

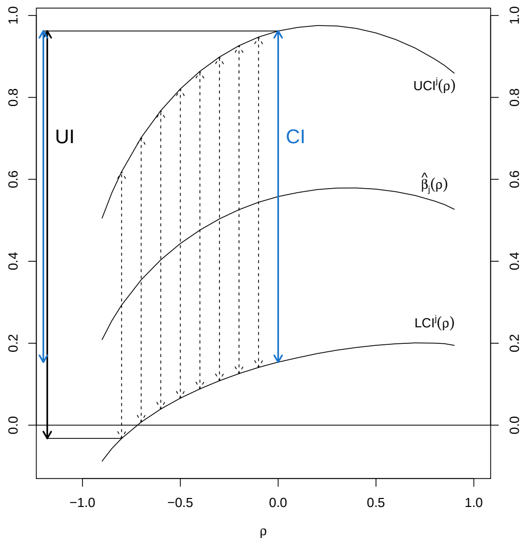

Figure 5: Sensitivity analysis ($\alpha = 5\%$, $\rho \in [-0.8, 0]$) for Obesity, Swedish men (see text).

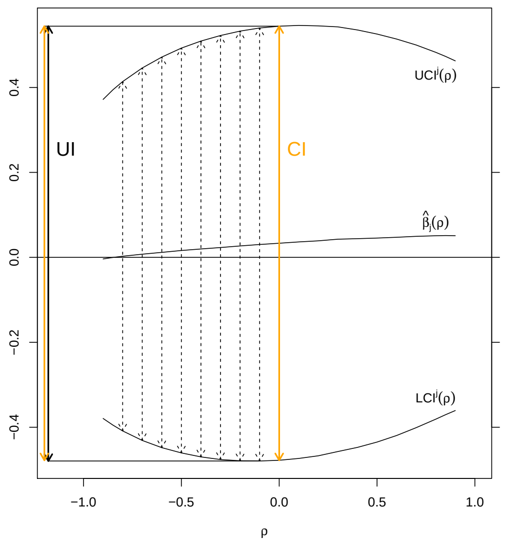

Figure 6: Sensitivity analysis ($\alpha = 5\%$, $\rho \in [-0.8, 0]$) for Obesity, Dutch men (see text).

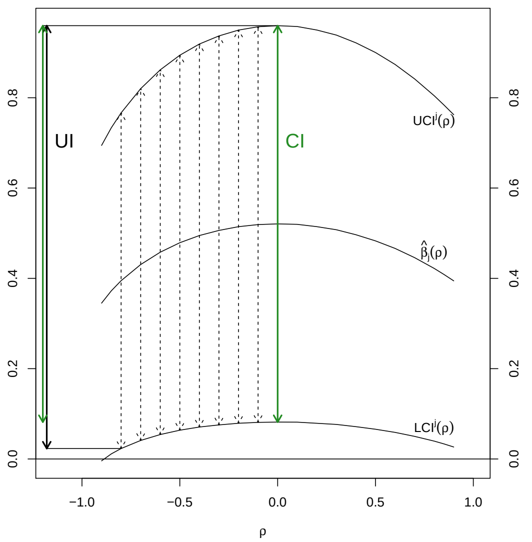

Figure 7: Sensitivity analysis ($\alpha = 5\%$, $\rho \in [-0.8, 0]$) for Obesity, Italian men (see text).

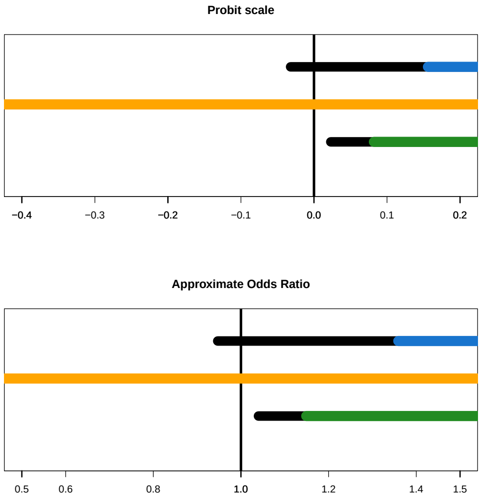

Figure 8: Confidence interval assuming ignorable dropout (blue Sweden, orange Netherlands and green Italy) drawn over the uncertainty interval assuming $\rho \in [-0.8, 0]$ in black.

observed patterns are possible. Those are (a) observed units with $Z = 0$, Y not observed (b) observed units with $Y = 0, Z = 1$ and (c) observed units with $Y = 1, Z = 1$. We have:

$$P(Z = 0|\mathbf{x}) = 1 - \Phi(\boldsymbol{\delta}\mathbf{x}),$$

$$P(Y = 0, Z = 1|\mathbf{x}) = \Phi_2(-\boldsymbol{\beta}\mathbf{x}, \boldsymbol{\delta}\mathbf{x}, -\rho),$$

$$P(Y = 1, Z = 1|\mathbf{x}) = \Phi_2(\boldsymbol{\beta}\mathbf{x}, \boldsymbol{\delta}\mathbf{x}, \rho),$$

where $\Phi(\cdot)$ is the standardized normal cdf while $\Phi_2(\cdot, \cdot; \rho)$ is the standardized bivariate normal cdf with correlation ρ. Using the notation in Greene (2012; p. 779-780), for each observation i, we let $q_i = 2y_i - 1$, $w_i = q_i\boldsymbol{\beta}^T\mathbf{x}_i$ and $\rho_i^* = q_i\rho$. The expressions above can be rewritten as:

$$P(Z, ZY|\mathbf{x}) = (1 - \Phi(\delta x_i))^{1-z_i}\, \Phi_2(w_i, \delta^T x_i, \rho_i^*)^{z_i}.$$

From this the log-likelihood can be derived:

$$\ell(\boldsymbol{\delta}, \boldsymbol{\beta}, \rho) = \sum_i (1 - z_i) \ln\{1 - \Phi(\boldsymbol{\delta}^T\mathbf{x}_i)\} + \sum_i z_i \ln\{\Phi_2(w_i, \boldsymbol{\delta}^T\mathbf{x}_i; \rho_i^*)\}. \tag{2}$$

In order to decrease computing time in the optimization of (2), we use the analytical first derivative of the log-likelihood function (gradient function):

$$\frac{d\ell(\boldsymbol{\delta}, \boldsymbol{\beta}, \rho)}{d\boldsymbol{\beta}} = \sum_i z_i \frac{d\ln\{\Phi_2(w_i, \boldsymbol{\delta}^T\mathbf{x}_i; \rho_i^*)\}}{d\boldsymbol{\beta}} = \sum_i z_i \frac{q_i\phi(\beta^T\mathbf{x})\Phi\left(\frac{\delta^T\mathbf{x} - \rho\beta^T\mathbf{x}}{\sqrt{1-\rho^2}}\right)}{\Phi_2(w_i, \delta^T\mathbf{x}_i; \rho_i^*)},$$

$$\begin{aligned}
\frac{d\ell(\boldsymbol{\delta}, \boldsymbol{\beta}, \rho)}{d\boldsymbol{\delta}} &= \sum_i (1 - z_i) \frac{d\ln\{1 - \Phi(\boldsymbol{\delta}^T\mathbf{x}_i)\}}{d\boldsymbol{\delta}} + \sum_i z_i \frac{d\ln\{\Phi_2(w_i, \boldsymbol{\delta}^T\mathbf{x}_i; \rho_i^*)\}}{d\boldsymbol{\delta}} \\
&= \sum_i (1 - z_i) \frac{-\phi(\boldsymbol{\delta}^T\mathbf{x}_i)}{1 - \Phi(\boldsymbol{\delta}^T\mathbf{x}_i)}\mathbf{x}_i^T + \sum_i z_i \frac{\phi(\boldsymbol{\delta}^T\mathbf{x})\Phi\left(q_i\frac{\boldsymbol{\beta}^T\mathbf{x} - \rho\boldsymbol{\delta}^T\mathbf{x}}{\sqrt{1-\rho^2}}\right)}{\Phi_2(w_i, \boldsymbol{\delta}^T\mathbf{x}_i; \rho_i^*)}\mathbf{x}_i^T.
\end{aligned}$$

References

Copas, J. and Li, H. (1997). Inference for non-random samples. Journal of the Royal Statistical Society: Series B 59(1), 55-95.

Demidenko, E. (2004) Mixed models theory and applications. John Wiley and Sons, Inc. Hoboken

Genbäck, M., Stanghellini, E. and de Luna, X. (2015) Uncertainty intervals for regression parameters with non-ignorable missingness in the outcome. Statistical Papers 56(3), 829-847.

Greene, W.H. (2012) Econometric analysis. 7 th edn. Pearson Education. Harlow

Stingo, F.C., Stanghellini, E. and Capobianco, R. (2011) On the estimation of a binary response model in a selected population. Journal of statistical planning and inference 141(10), 3293-3303.

Vansteelandt, S., Goetghebeur, E., Kenward, M.G. and Molenberghs, G. (2006) Ignorance and uncertainty regions as inferential tools in a sensitivity analysis. Statistica Sinica 16(3), 953-979.
